# Accident-Related Stress in the Elderly: A Review of Current Trends and Implications for Mental Health

**DOI:** 10.1192/j.eurpsy.2025.869

**Published:** 2025-08-26

**Authors:** A. Argyriadis, E. Kotrotsiou, S. Kotrotsiou, D. Katsarou, A. Patelarou, E. Patelarou, A. Argyriadi

**Affiliations:** 1 Nursing, Frederick University, Nicosia, Cyprus; 2 Nursing, University of Patras, Patra; 3 Department of Preschool Education Sciences and Educational Design, University of the Aegean, Rhodes; 4 Nursing, Hellenic Mediterranean University, Heraklion, Greece; 5 United Arab Emirates University, Al Ain, United Arab Emirates

## Abstract

**Introduction:**

Accidents, whether minor or severe, can have significant psychological impacts, especially in elderly populations. Stress related to accidents often exacerbates pre-existing conditions or leads to new mental health challenges such as anxiety, depression, or post-traumatic stress disorder (PTSD). The psychological impact of accidents on elderly individuals is often compounded by physical frailty, social isolation, and diminished coping mechanisms. Following an accident, elderly individuals may face prolonged recovery periods, limited mobility, and a reduced sense of independence, all of which can heighten stress levels. Additionally, the fear of future accidents may lead to avoidance behaviors, further isolating them from social interactions and routine activities, thus exacerbating anxiety and depression. Pre-existing mental health conditions, such as mild cognitive impairment or chronic illness, can worsen under accident-related stress.

**Objectives:**

This study aims to explore recent trends in understanding and addressing accident-related stress in elderly individuals, focusing on the psychological, social, and physiological factors contributing to their vulnerability. The primary objective of this study is to examine the psychological, social, and physiological factors that increase the vulnerability of elderly individuals to accident-related stress.

**Methods:**

A mixed-methods approach was used, combining a systematic review of literature from 2015 to 2024 and interviews with mental health professionals. The sample consisted of 30 peer-reviewed studies and 25 elderly individuals aged 65 and above who had experienced accidents within the last year. Studies were selected based on relevance to accident-related stress in the elderly, with an emphasis on post-accident psychological outcomes and interventions.

**Results:**

Results indicated that the elderly are more susceptible to prolonged stress responses following accidents due to physical fragility, social isolation, and reduced coping mechanisms. The review also highlighted an underutilization of mental health services in this demographic, despite the availability of stress-reduction programs. Furthermore, findings showed that older adults who participated in targeted mental health interventions, such as cognitive-behavioral therapy and peer support groups, experienced better outcomes in managing stress compared to those who did not.

**Conclusions:**

In conclusion, accident-related stress in the elderly presents unique challenges that require specialized attention. Healthcare providers should prioritize early identification and tailored interventions to mitigate the long-term psychological effects of accidents in this vulnerable population.

**Disclosure of Interest:**

None Declared

